# Effect of PEG Molecular Structure on Interfacial Stabilization and Laboratory Stability of High-Internal-Phase Emulsified Asphalt

**DOI:** 10.3390/ma19214468

**Published:** 2026-10-23

**Authors:** Hongliang Li, Lingyun Kong, Deyi Wang, Yujun Wang

**Affiliations:** 1 School of Civil Engineering, Chongqing Jiaotong University, Chongqing 400074, China; konglingyun@cqjtu.edu.cn (L.K.); deyi.wang@imdea.org (D.W.); 15803011141@163.com (Y.W.); 2 Xinjiang Communications Construction Group Co., Ltd., Urumqi 830009, China; 3 IMDEA Materials Institute, C/Eric kandel, 2, 28906 Getafe, Madrid, Spain

**Keywords:** high-internal-phase emulsified asphalt, molecular dynamics simulation, polyethylene glycol chain length, interfacial interactions, storage stability, thermal transition

## Abstract

High-internal-phase emulsified asphalt (HIPEA), characterized by a high asphalt volume fraction, may reduce water demand relative to conventional emulsified asphalt. However, the relationship between polyethylene glycol (PEG) molecular structure, asphalt–water interfacial organization, and laboratory stability remains incompletely quantified. This study compared four model systems containing octadecyltrimethylammonium chloride (STAC) alone or STAC combined with PEG with ethylene oxide (EO) numbers of 22, 45, and 55 (P22, P45, and P55). Molecular dynamics (MD) simulations were combined with dispersion observations, fluorescence microscopy, storage monitoring, centrifugation, differential scanning calorimetry (DSC), and exploratory Pearson correlation analysis. The results showed that STAC alone did not produce a uniform emulsion under the tested preparation conditions, whereas the PEG-containing formulations formed oil-in-water structures. The initial mean droplet diameter decreased from 4.20 μm for P22 to 2.57 μm for P55. After 22 days, the mean diameters were 6.70, 6.20, and 5.42 μm for P22, P45, and P55, respectively, indicating less pronounced droplet growth for the longer PEG formulations under the tested storage conditions. Under the same centrifugation protocol, the reported separated continuous-phase mass decreased from 0.233 g for P22 to 0.059 g for P55, corresponding to a 74.7% reduction based on these mass values. The characteristic DSC peak temperature increased from 49.853 °C for P22 to 61.105 °C for P55; this shift indicates a change in thermal response but does not, by itself, establish improved overall thermal stability. The MD results suggested differences in interfacial organization among the constructed systems. Pearson analysis identified exploratory associations between molecular descriptors and macroscopic indicators, but its interpretation is limited by the four-system dataset and the lack of strict compositional normalization. Overall, the findings provide molecular-level insights into the tested STAC/PEG systems and their laboratory stability. Further composition-controlled simulations and pavement-related testing are required before drawing conclusions about field performance.

## 1. Introduction

Emulsified asphalt is used mainly in pavement maintenance and preservation operations, including surface treatments, tack coats, and cold or in-place recycling, because it can be handled at lower temperatures than hot-mix asphalt [1,2]. Product classification and specification depend on the jurisdiction; in Europe, EN 13808 provides a framework for specifying cationic bituminous emulsions. Conventional colloid-milled emulsified asphalt commonly contains about 60–70% asphalt by mass and often has droplet sizes of approximately 5–50 μm, although these ranges depend on formulation and processing [3,4]. Compared with these conventional systems, high-internal-phase emulsified asphalt (HIPEA) is designed with an asphalt dispersed phase volume fraction above approximately 74%. Its high dispersed phase fraction and closely packed droplets may reduce water demand, but they also make interfacial film integrity and storage stability particularly important [5]. The present study addresses laboratory emulsion formation and stability, not the performance of pavement mixtures.

Previous studies have explored the preparation and performance of high-asphalt-content and high-internal-phase bitumen emulsions. In terms of the process mechanism research, Arenas Calderon Edward et al., through microscopic observation and kinetic analysis, revealed the microstructure evolution laws during the phase transformation of emulsified asphalt, discovering that the system underwent a transformation from multiple emulsions to monodisperse emulsion, and observed four characteristic stages on the viscosity change curve [6,7]. The Gutierrez research group systematically investigated the influence of different process parameters on product performance and focused on the regulatory effects of key factors such as mixing time, initial volume fraction, and stirring rate on the final emulsion droplet size [8]. Recent studies have further shown that droplet size and size distribution strongly influence the rheological behavior and storage stability of high-asphalt-content emulsified asphalt [9,10]. At the molecular scale, molecular dynamics (MD) simulation has emerged as an effective approach for elucidating asphalt–emulsifier interfacial interactions. Previous studies have examined the effects of emulsifier type, hydrophilic group structure, functional group position, alkyl chain length, and inorganic ions on interfacial energy, molecular diffusion, demulsification behavior, and asphalt–aggregate adhesion [11,12].

Previous studies have mainly focused on the preparation process, droplet morphology evolution, rheological behavior, and empirical stability evaluation of high-asphalt-content emulsions. Although molecular dynamics simulations have provided valuable insights into asphalt–emulsifier interactions, most studies have concentrated on conventional emulsified asphalt systems. The synergistic effects between multiple stabilizing components and their influence on HIPEA interfacial stability remain insufficiently understood. In particular, the relationship between PEG molecular architecture, interfacial interactions, and macroscopic stability performance has not been systematically established.

The objective of this study is to compare four systems—STAC, STAC+P22, STAC+P45, and STAC+P55—using MD simulation, dispersion observations, fluorescence microscopy, storage monitoring, centrifugation, DSC, and exploratory correlation analysis. Specifically, the study examines (i) the simulated distribution and interactions of components near the asphalt–water interface, (ii) changes in droplet size and phase separation under the laboratory protocols used, and (iii) whether the measured molecular descriptors co-vary with the observed laboratory indicators. The study does not claim to establish pavement-level performance or a causal effect of PEG chain length independently of all composition variables.

## 2. Simulation and Experimentation

### 2.1. Raw Materials

**Figure 1:**
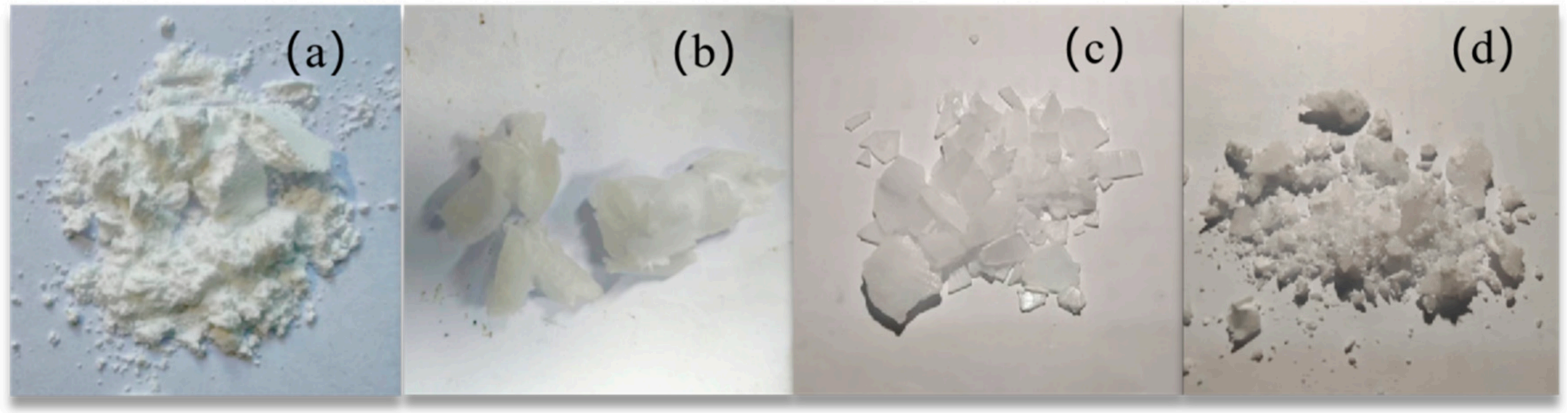
Various high-internal emulsifiers. (**a**) STAC, (**b**) p22, (**c**) p45 and (**d**) p55.

The asphalt used was Kunlun 70# base asphalt, paroduction of China Xinjiang Karamay Petrochemical Company. Its penetration, softening point, and ductility are reported in Table 1. The cationic surfactant used was octadecyltrimethylammonium chloride (the abbreviation is STAC; industrial grade; molecular formula C_21_H_46_ClN; molecular weight 348.05 g/mol), the physical object of STAC is shown in Figure 1a. The polymer was polyethylene glycol methyl ether (PEG), represented by P22, P45, and P55 with nominal EO numbers of 22, 45, and 55, respectively, the physical samples of P22, P45, and P55 are shown in Figure 1b–d. The manuscript uses “STAC” as the supplier/product abbreviation for octadecyltrimethylammonium chloride; this abbreviation is retained consistently throughout. The testing of asphalt indicators follows the JTG 3410-2025 standard and test methods. The determination method of the four components of asphalt adopts the DS method specified in T0618.

### 2.2. Model Construction and Validation

**Figure 2:**
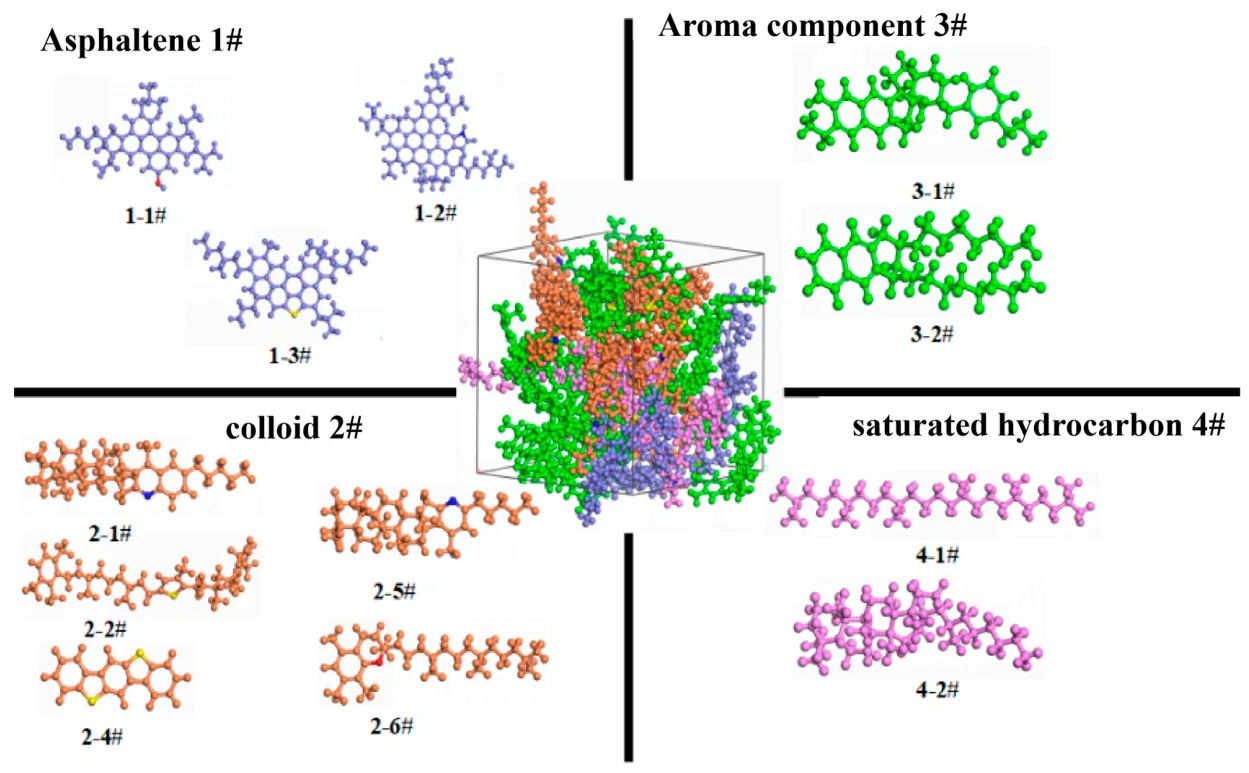
Twelve-molecule model("# " indicates the molecular number).

In this study, Kunlun 70# base asphalt was selected, and the proportions of its four SARA fractions are presented in Table 2. Based on these fractions, the twelve-molecule asphalt model developed by Liang Li and Matthew L. Greenfield was employed, as shown in Figure 2. The model uses twelve representative molecular structures to characterize the chemical characteristics of the saturates, aromatics, resins, and asphaltenes fractions [13].

**Figure 3:**
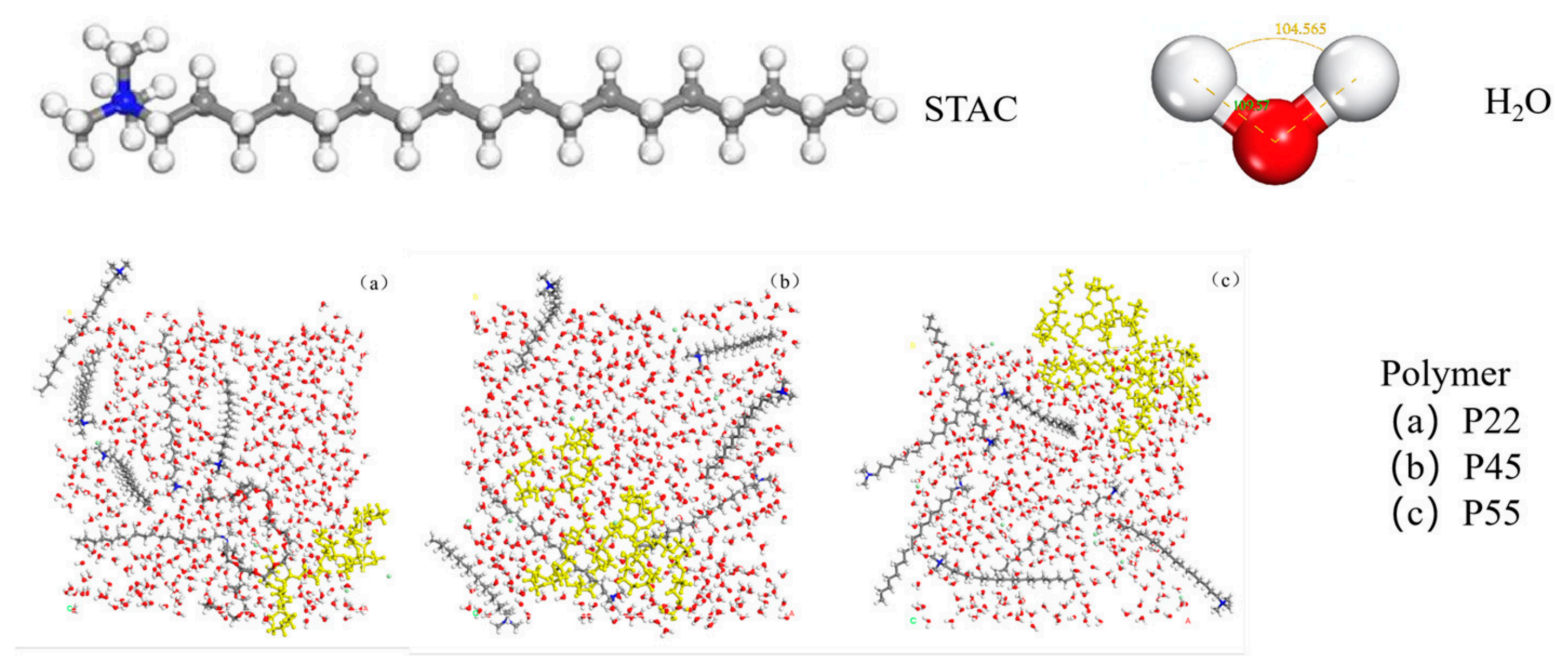
Molecular model of STAC/H_2_O/PEG.

The emulsifier was octadecyltrimethylammonium chloride (STAC), a cationic quaternary ammonium surfactant. The abbreviation STAC is used here as the material identifier throughout the manuscript. PEG is represented by three nominal EO chain lengths, P22, P45, and P55. The ether oxygen atoms of PEG can interact with water molecules, contributing to the interfacial hydration environment. However, the present analysis does not directly quantify PEG–water hydrogen-bond counts, so hydration interactions are discussed as a plausible interpretation of the structural observations rather than as a measured hydrogen-bond network. The molecular model of STAC/H_2_O/PEG is shown in Figure 3.

**Figure 4:**
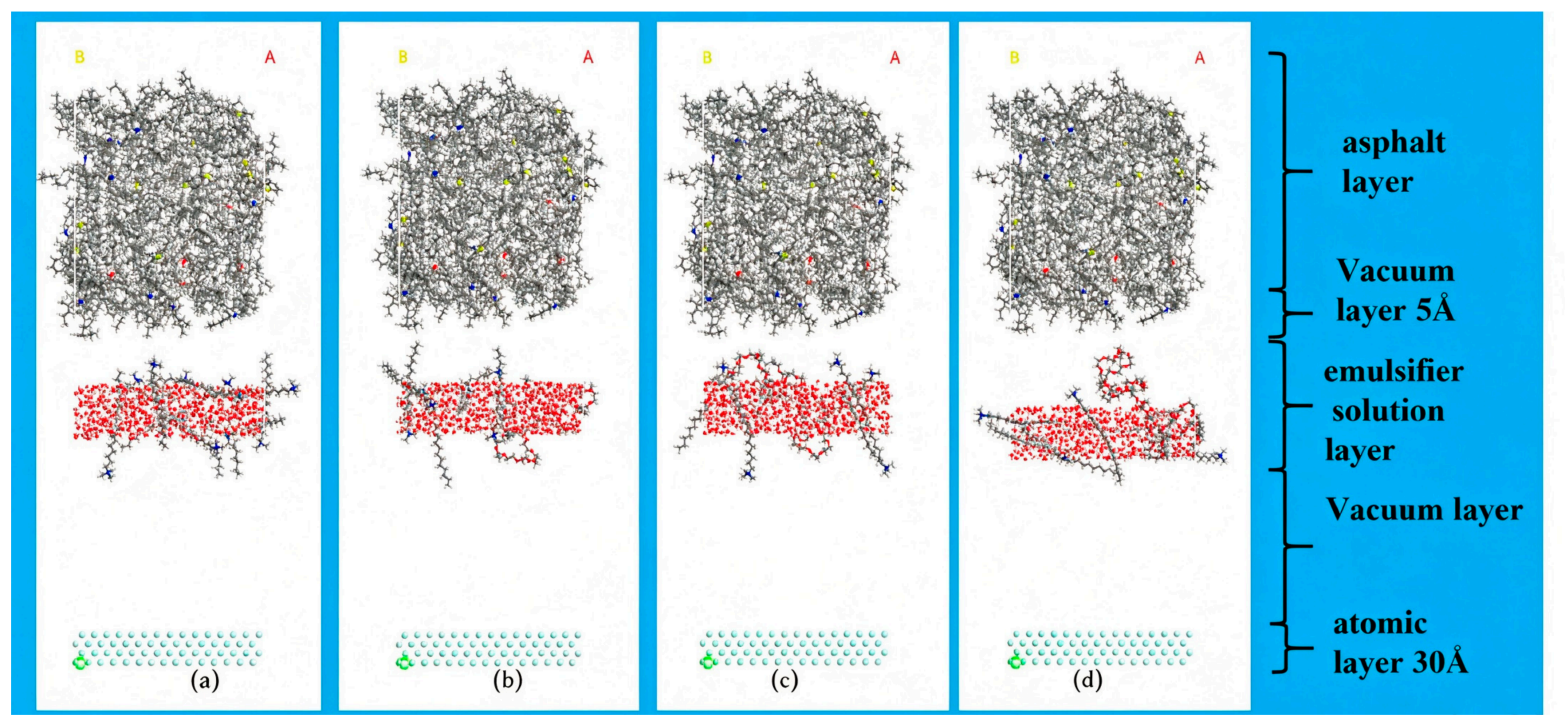
Schematic diagrams of four molecular models of HIPEA: (**a**) STAC, (**b**) STAC+P22, (**c**) STAC+P45 and (**d**) STAC+P55.

For the experimental formulations, the asphalt content was set to 80 wt% of the total formulation, while the remaining components were added according to the mass ratios listed in Table 4. The soap solution was prepared using water: STAC: PEG at a mass ratio of 8:1:1. These experimental mass-based formulation ratios are distinct from the simplified molecular composition used in the MD models and should not be interpreted as directly equivalent. In the MD systems, molecule counts were selected to construct tractable asphalt–water–STAC/PEG interfaces; because PEG molecular weights and molecule counts differed, the models do not constitute a strictly normalized single-variable comparison. The component counts and dimensions are reported in Table 3. The schematic diagrams of the four molecular models of HIPEA are shown in Figure 4. A 30 Å vacuum layer was introduced along the z direction, and a 5 Å separation was maintained between the asphalt and emulsifier layers to reduce artificial interactions between periodic images and allow an interface to form [14]. Periodic boundary conditions were applied in the x and y directions [15]. These models are simplified interfacial representations and cannot reproduce the complete composition or microscale morphology of experimental HIPEA; therefore, the simulation results are interpreted as qualitative molecular-level evidence rather than a quantitative reproduction of the experimental emulsion.

The initial density of each simulation box was set to 1.0 g cm^−3^ and subsequently equilibrated during isothermal–isobaric (NPT) simulations [16]. The simulation procedure consisted of four steps: (i) energy minimization for 50,000 steps; (ii) annealing in the NPT ensemble at 393 K for 200 ps over 20 cycles; (iii) further annealing in the canonical (NVT; constant number of particles, volume, and temperature) ensemble for 200 ps over 20 cycles; and (iv) equilibration for 500 ps in the NPT ensemble followed by a 500 ps NVT production trajectory. The NPT stage was used primarily for density equilibration, whereas the final NVT trajectory was used for structural and interfacial analyses. A 500 ps production trajectory may not fully capture slow asphalt and PEG rearrangements; the results are therefore used for comparative analysis under the selected simulation conditions, not as proof of complete long-time equilibrium. The equilibrium assessment should be based on the density, temperature, and energy trajectories of all four systems. Model reliability was further assessed by comparing calculated solubility parameters and interfacial energies with values reported in the literature [17].

**Figure 5:**
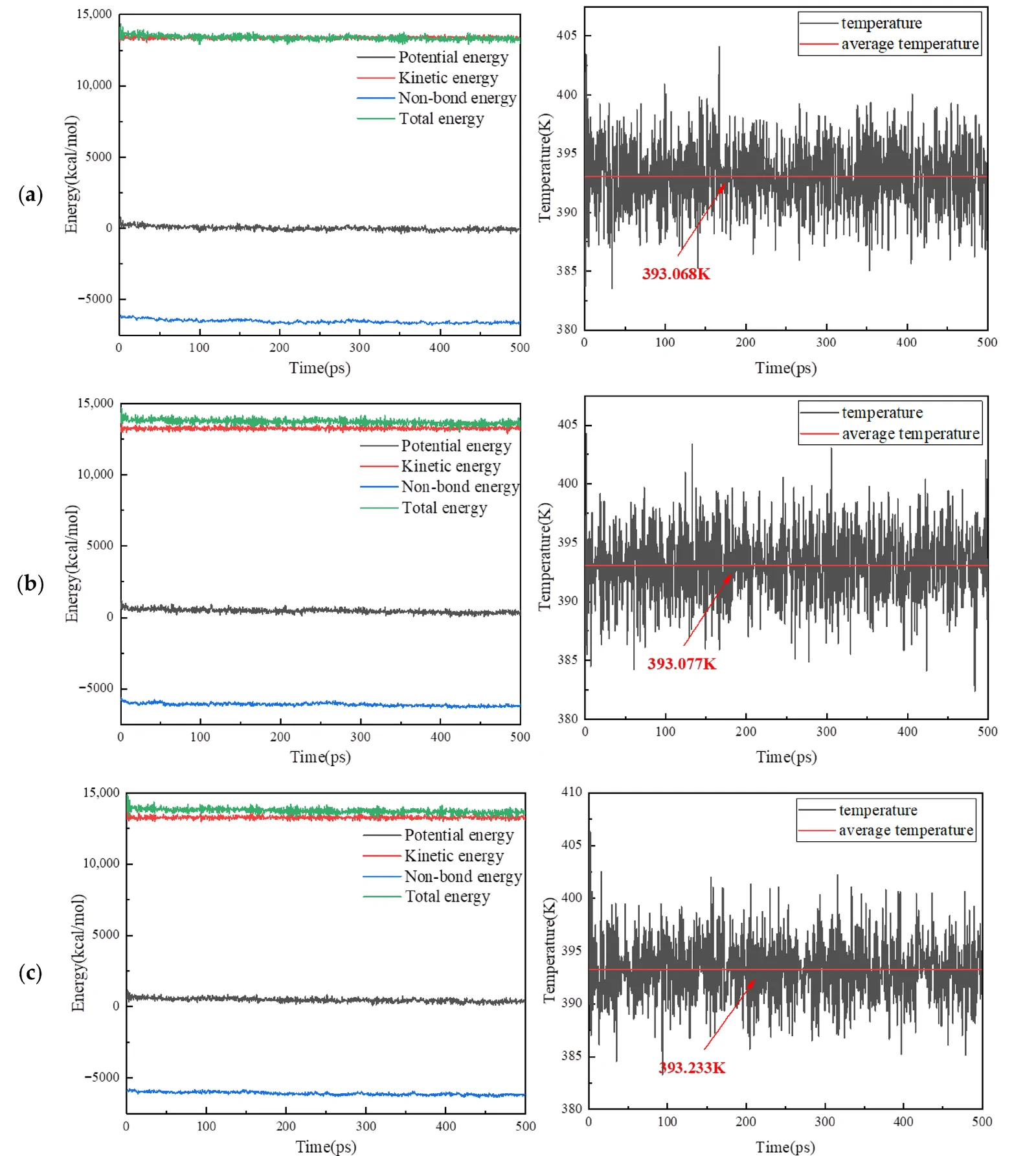

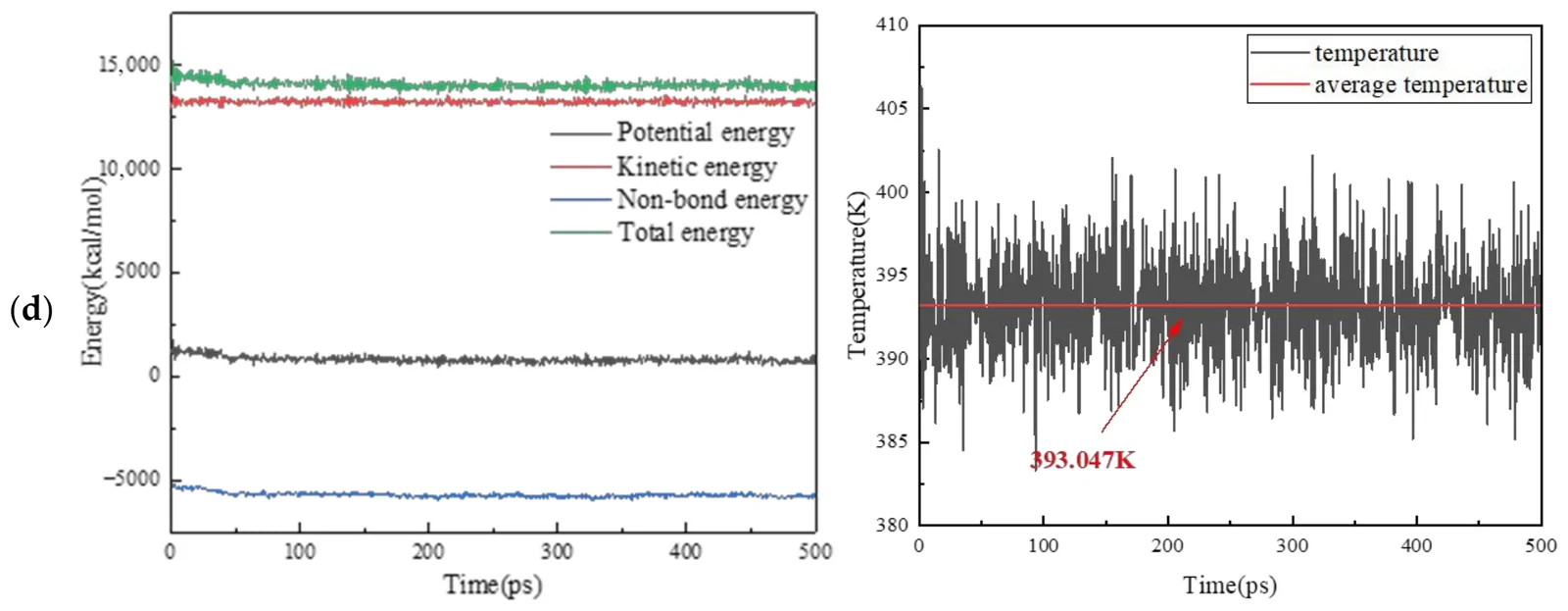
The energy and temperature changes in four asphalt models, (**a**) STAC, (**b**) P22 and STAC, (**c**) P45 and STAC, (**d**) P55 and STAC.

The average temperatures of the four systems were 393.068 K (STAC), 393.077 K (STAC+P22), 393.233 K (STAC+P45), and 393.047 K (STAC+P55), all close to the target temperature of 393 K. These average values indicate that the target temperature was maintained on average; the temperature fluctuation ranges and equilibration behavior should be evaluated from the full trajectories for all four systems. Figure 5 should not be interpreted as demonstrating equilibration of all systems unless the corresponding traces are shown.

### 2.3. HIPEA Preparation and Testing Methods

#### 2.3.1. HIPEA Preparation Method

**Figure 6:**
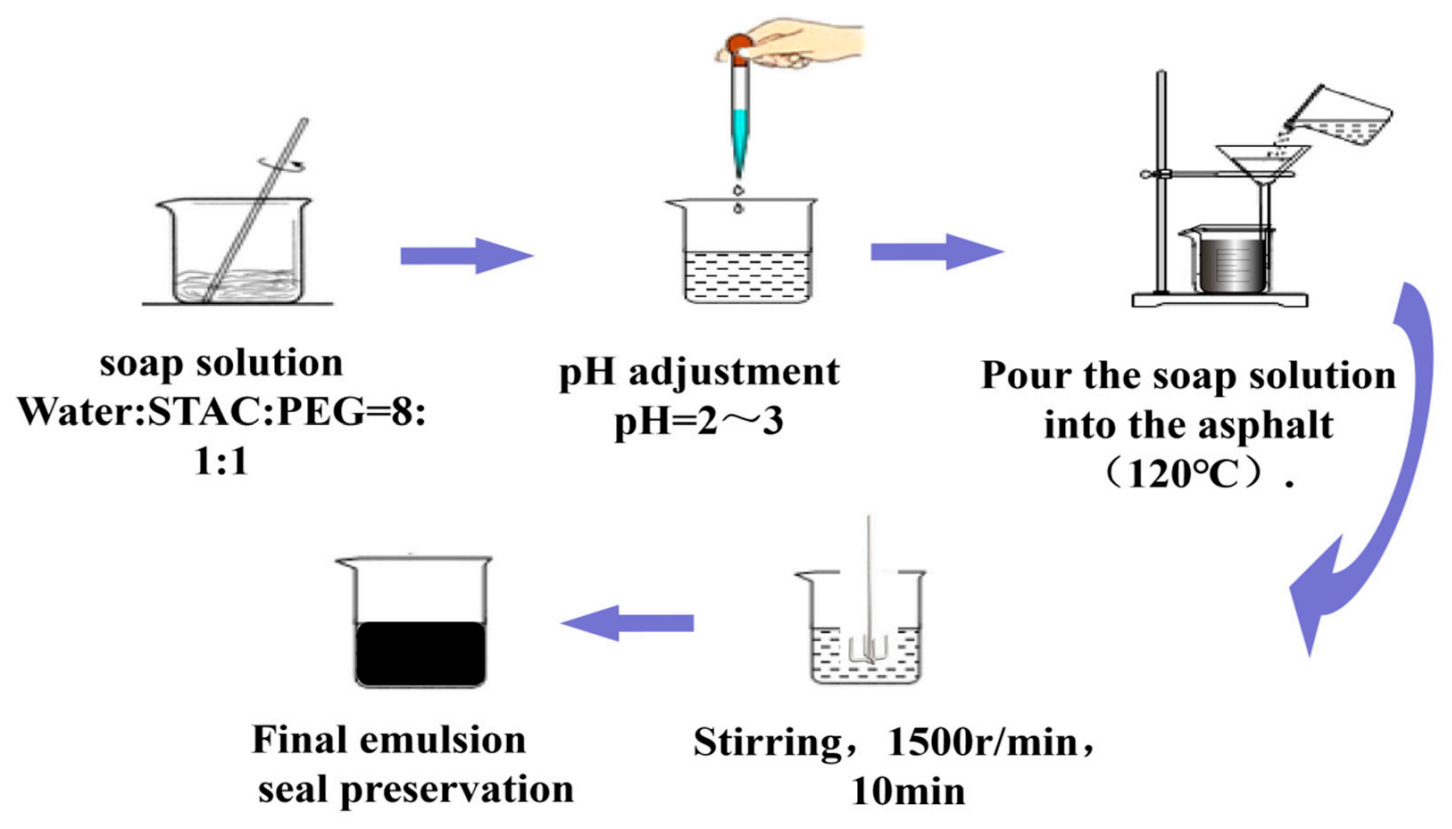
The preparation process flow of HIPEA.

High-internal-phase emulsified asphalt was prepared using an IA-RW20 digital stirrer. The target internal asphalt phase mass fraction was 80%, and the concentration of the soap solution was 20%. In the asphalt emulsion without PEG, the soap solution consists of water and STAC. In the STAC + P22/P45/P55 emulsion system, water, STAC, and PEG were mixed at a mass ratio of 8:1:1 to prepare the soap solution. The soap solution needs to be adjusted to a pH value of 2–3 using hydrochloric acid. The composition and mass ratio of the sample materials are shown in Table 4. The preparation procedure is shown in Figure 6.

#### 2.3.2. HIPEA Macroscopic Experiment Plan and Test Method

**Figure 7:**
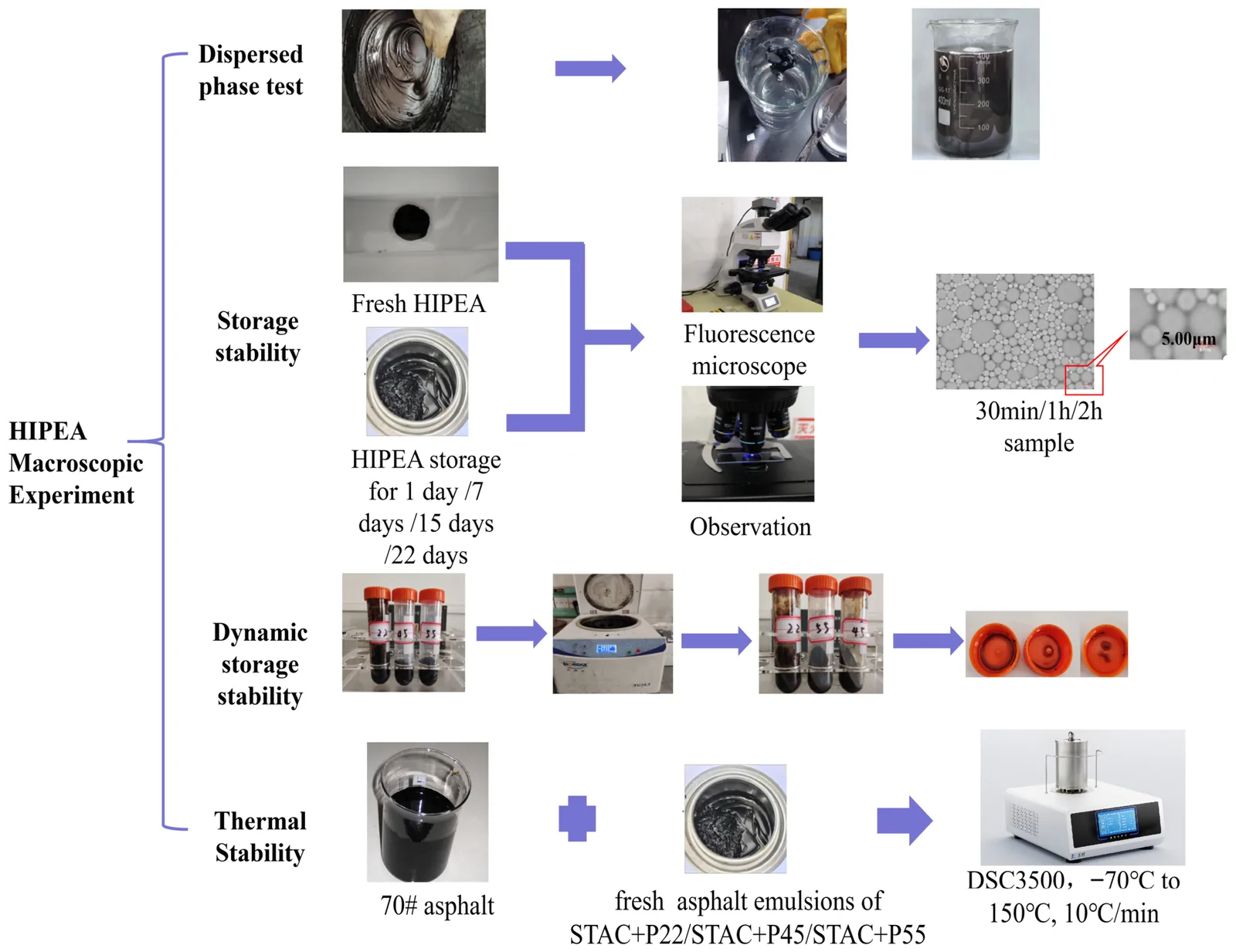
HIPEA macroscopic experiment plan.

HIPEA macroscopic experiments include the dispersed phase test, storage stability and thermal stability. The experimental plan is shown in Figure 7.

Storage stability involves storing freshly prepared emulsion samples at room temperature for varying durations (1, 7, 15, and 22 days). Microscopic examination of the samples’ morphology and droplet size distribution was performed using a fluorescence microscope (Nikon Eclipse Ci). Each sample was repeated 3 times; each image should count no less than 100 to 300 droplets.

(1) HIPEA dispersion test

Dispersion was assessed by adding a small amount of sample to distilled water and stirring for 1 min. Uniform dispersion was taken as qualitative evidence of emulsification, whereas visible agglomeration or floating indicated incomplete emulsification. This is a qualitative screening test and does not independently quantify long-term stability.

(2) Fluorescence microscopy test

A small amount of HIPEA was placed on a glass slide, covered with a coverslip, and heated at 80 °C for 3 min before fluorescence microscopy. The same preparation protocol was applied to all samples to support relative comparisons. However, no dedicated calibration experiment was performed to quantify water loss or compare droplet sizes before and after heating. Heating and coverslip pressure may therefore alter the observed morphology; the images are interpreted as comparative observations under the specified preparation conditions, not as an undisturbed measurement of the original emulsion structure [18].

(3) Differential scanning calorimetry (DSC)

DSC measurements were performed using a NETZSCH DSC 3500 differential scanning calorimeter to evaluate the thermal characteristics of 70# base asphalt and HIPEA systems with different EO numbers. Approximately 10–15 mg of sample was sealed in an aluminum crucible and heated from −70 to 150 °C at 10 °C/min under a high-purity nitrogen flow of 50 mL/min. The characteristic thermal parameters, including enthalpy change (ΔH), glass transition temperature (Tg), and characteristic transition temperatures, were determined from the DSC curves based on the peak temperature, peak width, and integrated peak area [19].

(4) Centrifugal stability test

The centrifugal stability of HIPEA systems with different PEG chain lengths was evaluated using a Lu Xiangyi TG18.5 benchtop centrifuge. Approximately 10 g of each sample was placed in a 50 mL centrifuge tube and centrifuged at 8000 rpm for 1 h. The rotor radius was reported as 8 cm, corresponding to a calculated relative centrifugal force of approximately 5724× *g*. After centrifugation, the separated continuous phase was removed and weighed. Each sample was tested in triplicate, and the mean was used for subsequent analysis. The calculation of separation percentage requires the initial sample mass and the exact calculation equation; these should be reported with replicate-level values and uncertainty if the original records are available [20].

## 3. Results and Analysis

### 3.1. Analysis of Surface Mass Transfer Behavior of HIPEA

#### 3.1.1. Molecular Rearrangement and Interfacial Assembly Behavior

**Figure 8:**
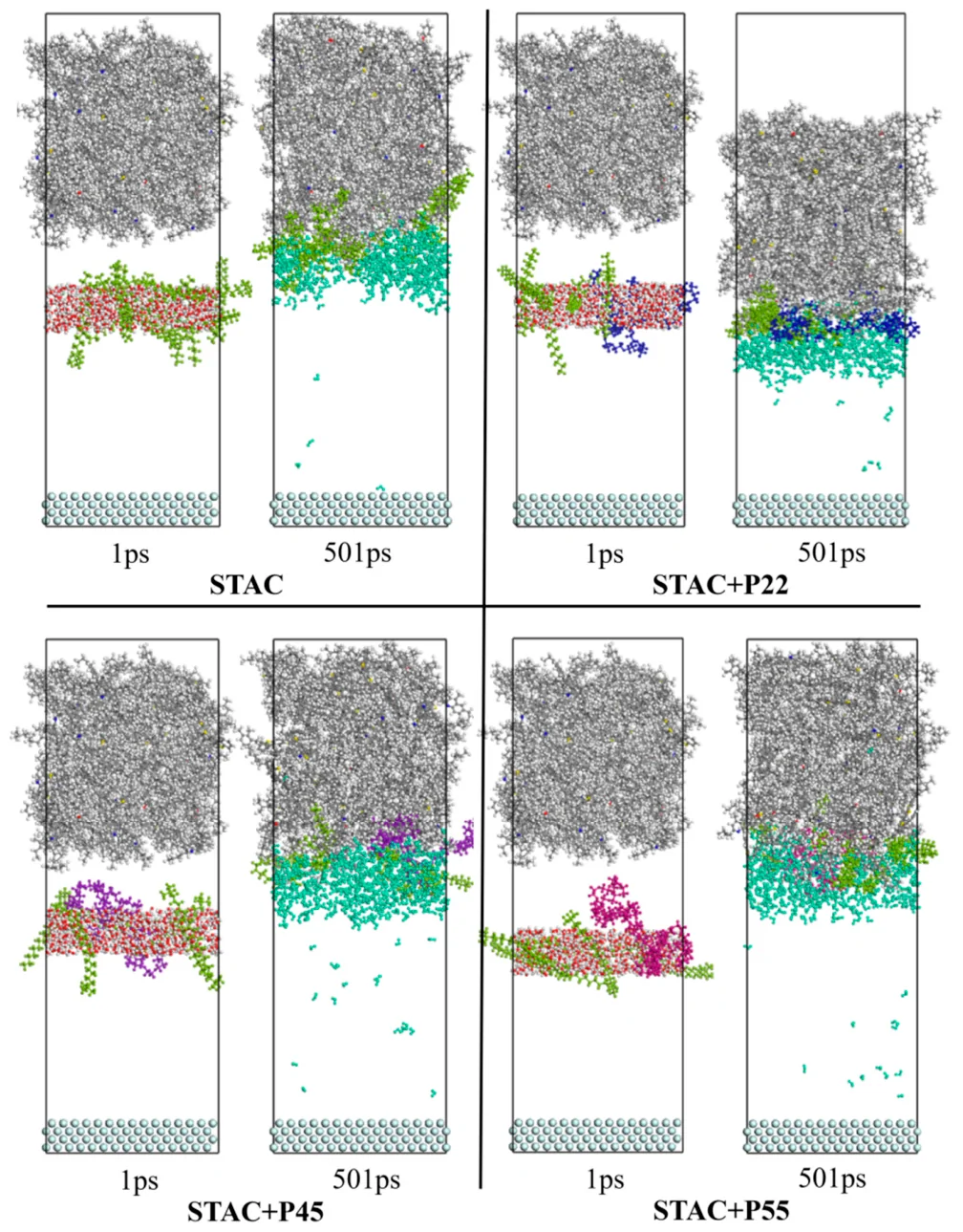
The molecular motion trajectories of four asphalt systems.

This study investigates the dynamic aggregation behavior of STAC and PEG at the oil–water interface by analyzing the molecular movement trajectories of asphalt, water, emulsifier and polymer molecules. As shown in Figure 8, it depicts the molecular motion trajectories of four HIPEA systems during their initial stage (1 ps) and equilibrium stage (501 ps).

At 1 ps, the components are randomly distributed, with no well-defined interfaces formed among asphalt, water, STAC, and PEG. Considerable intermolecular voids are observed, indicating that the system is still far from its equilibrium configuration. At 501 ps, substantial molecular rearrangement has occurred, accompanied by progressive interfacial formation and densification of the system. STAC has a C18 hydrophobic chain and a N+(CH_3_)_3_ hydrophilic head group. The C18 alkyl chain inserts into the asphalt phase; the positively charged hydrophilic head faces the water phase; a layer arrangement is formed at the oil–water interface. PEG migrates from the water phase to the interface; the chain segments interpenetrate in the STAC interface layer; the long-chain PEG (P55) has a larger coverage area.

Compared with the STAC system, the incorporation of PEG promoted a more compact interfacial structure. The hydrophobic alkyl chains of STAC interact with asphalt molecules through van der Waals forces, whereas the hydrophilic segments extend toward the aqueous phase. The PEG chains were distributed within the interfacial region and interacted with water molecules through ether oxygen-mediated interactions, contributing to the stabilization of the interfacial hydration layer.

Among the three PEG-containing systems, STAC+P55 exhibited the densest interfacial molecular arrangement. The longer PEG chains provided more ether oxygen sites and larger spatial coverage, facilitating stronger interactions with water molecules and improving the integrity of the interfacial hydration layer. In contrast, the STAC system showed insufficient interfacial organization due to the absence of polymer reinforcement, which explains its poor emulsification stability observed experimentally. These results indicate that PEG chain length plays a crucial role in regulating molecular assembly and interfacial stabilization of HIPEA.

#### 3.1.2. Radial Distribution Function Analysis

**Figure 9:**
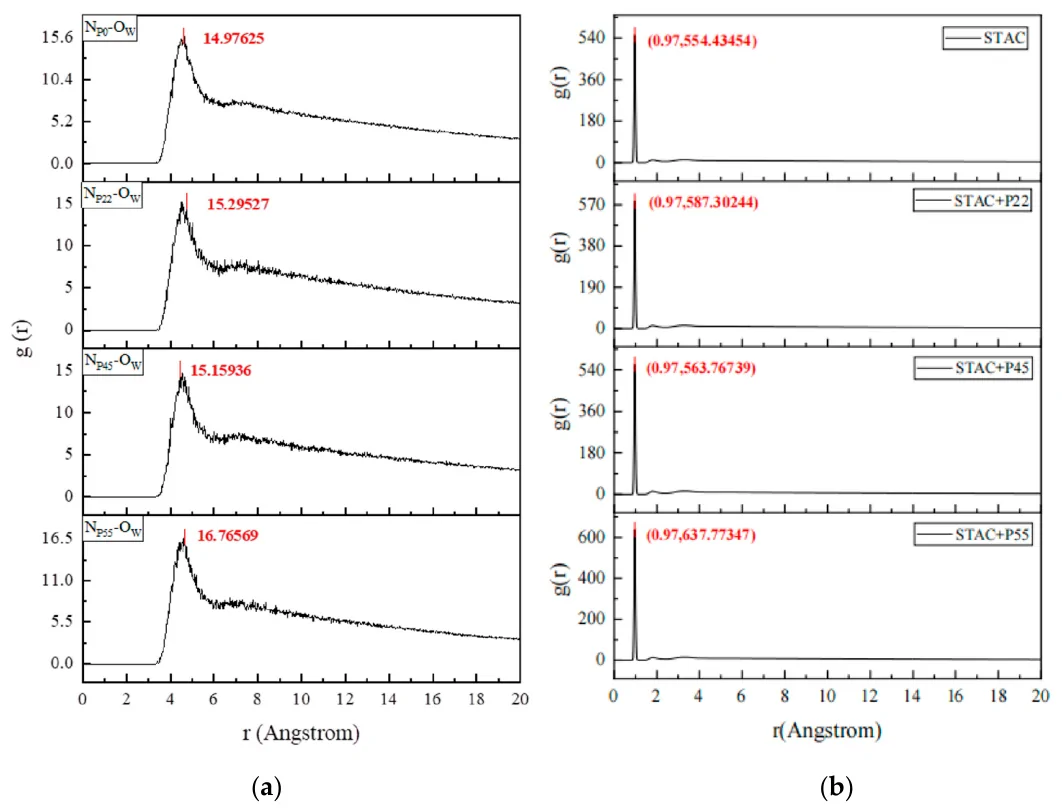
Radial distribution functions (**a**) N_stac_-O_w_, (**b**) O_W_-H_W._

The radial distribution function is a valuable tool for examining spatial correlations and interactions among distinct atoms or groups within a given system [21]. Figure 9 illustrates the RDFs for N atoms within the hydrophilic segment of STAC and O atoms in water molecules (N_stac_-O_w_), O atoms paired with H atoms in water molecules, and g(r) represents the probability of the appearance of the target atom/particle at the position r.

Figure 9a presents the RDF curves between the hydrophilic nitrogen atoms of STAC and oxygen atoms of water molecules (N_stac_-O_w_). All systems exhibited a distinct peak at approximately 4.5–5.0 Å, indicating preferential adsorption and aggregation of water molecules around the hydrophilic groups of STAC. A secondary peak appeared at approximately 7.0–7.5 Å, suggesting the formation of an extended hydration structure near the oil–water interface. The presence of these characteristic peaks indicates the multilayer organization of interfacial water molecules, which contributes to the construction of a stable hydrated interface [22].

Figure 9b shows the RDF between oxygen and hydrogen atoms of water molecules (O_W–H_W). The peak intensity increased after PEG incorporation, following the order STAC+P55 > STAC+P22 > STAC+P45 > STAC.

This result indicates that PEG may help maintain a more stable interfacial structure. PEG does not possess a conventional hydrophobic tail. Instead, the ether oxygen groups of PEG can interact with surrounding water molecules, while the polymer chains may contribute to interfacial stabilization through hydration effects and steric hindrance. Together with STAC adsorption at the asphalt–water interface, PEG may help maintain a more stable interfacial structure [23].

#### 3.1.3. Interfacial Formation Energy Analysis

Interfacial formation energy (Δ*E*) was calculated to quantify the interaction strength between the asphalt phase and the emulsifier-containing aqueous phase [24]. The interface can calculate the formula as follows:

(1) $$\Delta E=E_{\mathrm{total}}-(E_{\mathrm{Asp}}+E_{\mathrm{Emul}})$$

In the equation, *E*_total_ denotes the overall molecular potential energy (kcal/mol) of the emulsified asphalt oil/water system; *E*_Emul_ represents the molecular potential energy (kcal/mol) of the emulsifier solution system; and *E*_Asp_ signifies the cumulative potential energy (kcal/mol) of asphalt molecules. The parameter Δ*E* denotes the energy required for emulsifier molecules to reduce the interfacial tension at the oil–water interface; hence Δ*E* is negative. A negative Δ*E* value implies that the emulsifier molecules are adsorbing at the interface between the asphalt and water phases. Furthermore, a higher absolute value of Δ*E* indicates stronger adsorption between the two phases, enhancing the emulsifier’s capacity to reduce interfacial tension in the asphalt/water system, thereby favoring the formation of a monolayer film at the interface. Consequently, the overall stability of the emulsified asphalt solution system is increased [25]. Conversely. When Δ*E* ≥ 0, it suggests that the emulsifier is not adsorbing at the interface between the two phases, or that the interaction between them is minimal, rendering the entire system unstable [26]. The computational outcomes are detailed in Table 5.

Table 5 reveals that all four emulsified asphalt systems exhibit negative Δ*E* values, suggesting mutual adsorption of the emulsifier at the asphalt–water interface. Comparative analysis of ΔE magnitudes indicates that |Δ*E*|_STAC+P55_ > |Δ*E*|_STAC+P45_ > |Δ*E*|_STAC+P22_ > |Δ*E*|_STAC_. It is indicated that the introduction of polymer (PEG) significantly enhances the adsorption efficiency of the emulsifier at the interface. This improvement can be attributed to the dual stabilization effect of PEG: the polymer chains increase interfacial coverage and simultaneously enhance interactions with water molecules through hydrated ether oxygen groups. The STAC+P55 system exhibited the strongest interfacial interaction (Δ*E* = −326.359 kcal/mol), indicating that longer PEG chains promote the formation of a more stable interfacial film under the investigated formulation conditions.

#### 3.1.4. Solubility Parameters Analysis

**Figure 10:**
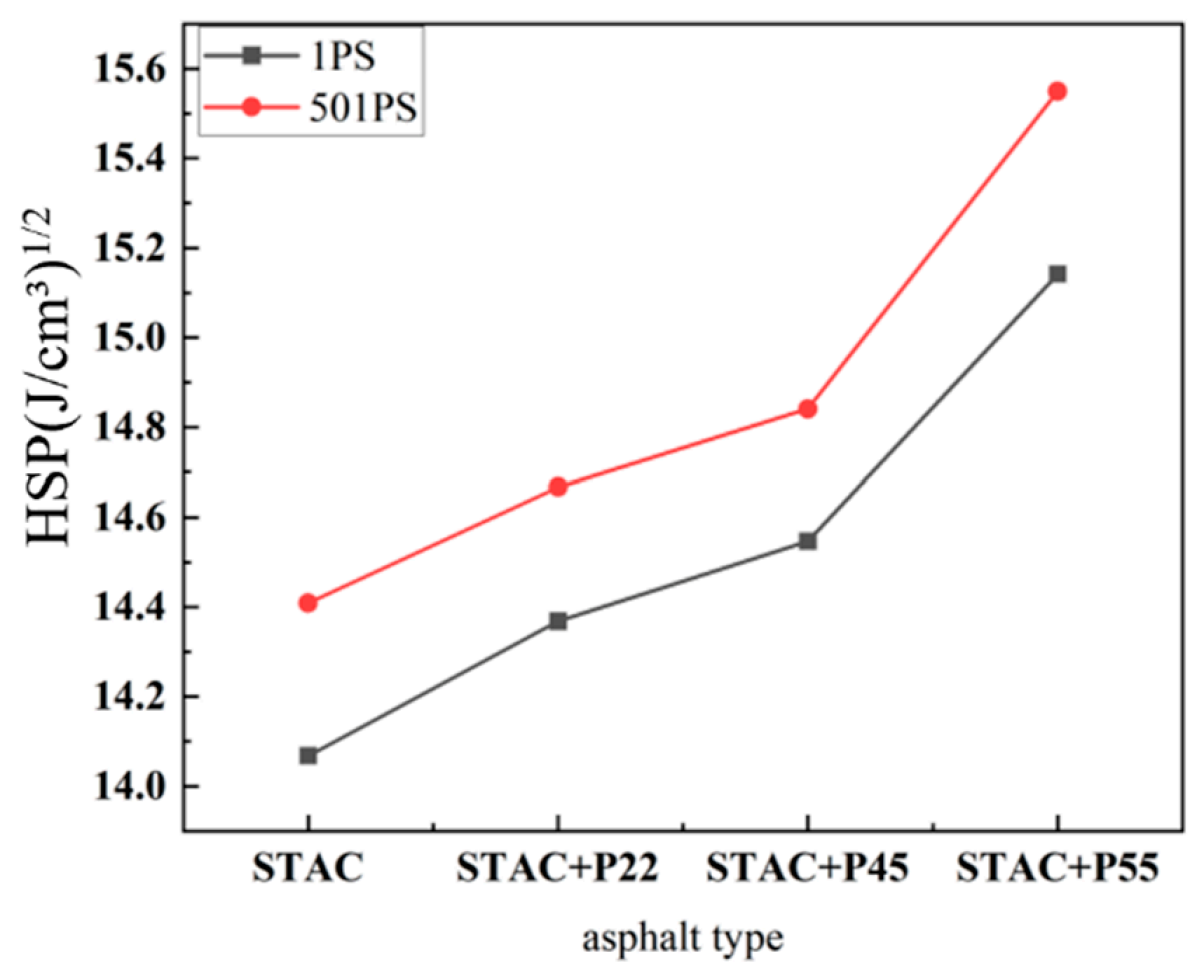
Solubility parameters of the HIPEA system at 1 ps and 501 ps.

**Figure 11:**
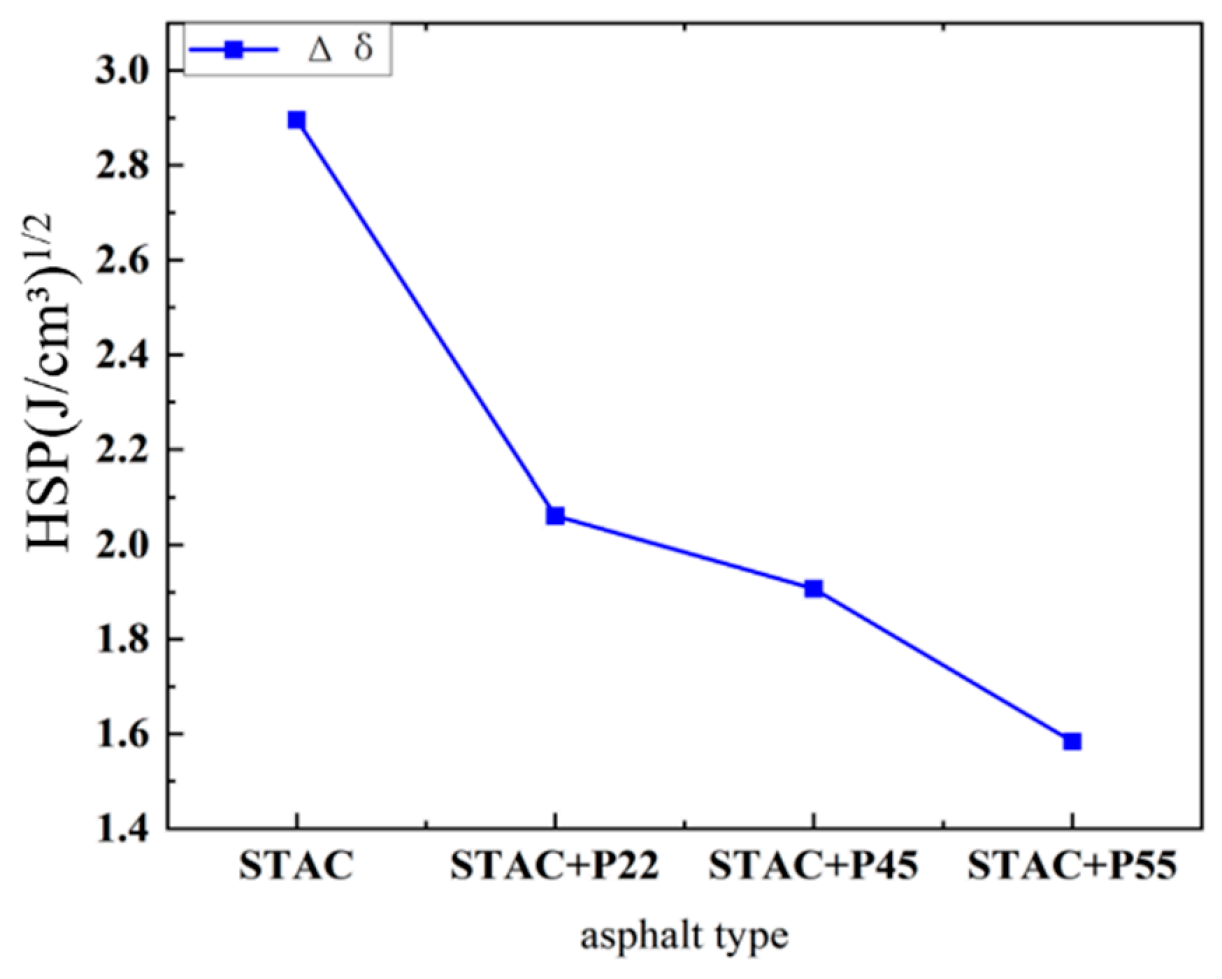
Solubility difference between four high-internal-phase emulsifier solutions and asphalt molecules.

The solubility parameter was analyzed to evaluate the compatibility between the asphalt phase and the emulsifier solution [27]. The closer the solubility parameter values Δδ between two entities, the higher their compatibility [28]. Detailed findings are presented in Figure 10 and Figure 11.

Figure 10 compares the Hildebrand solubility parameters (HSP) of the systems at the initial stage (1 ps) and the equilibrium stage (501 ps). At 501 ps, the SP values across all systems are markedly higher than at 1 ps, indicating that molecular mixing has deepened and intermolecular interactions have intensified as the systems approach equilibrium. Notably, the data at 501 ps represent the average values over the last 100 ps of the equilibrium trajectory (401–500 ps) rather than a single instantaneous frame, thereby minimizing the inherent fluctuations of molecular dynamics simulations. This increase in SP reflects a more homogeneous and stable molecular distribution during the emulsification process.

Figure 11 reveals that all systems exhibit Δδ values ranging from 1.6 to 2.9 (J/cm^3^)^1/2^, notably below the critical threshold of 4.1, confirming favorable compatibility across all systems. Particularly noteworthy is that systems incorporating high-molecular-weight polymers display lower Δδ values than pure STAC systems. Under the studied formulations and preparation conditions, Δδ decreased as the polyethylene glycol chain length increased, with STAC+P55 exhibiting the lowest value. The addition of PEG can enhance the compatibility between the emulsifier solution and the asphalt. Moreover, the elongation of the polyethylene glycol chain correlates with a more pronounced enhancement effect, facilitating the amalgamation of oil and water phases and bolstering the stability of the high-internal-phase emulsion.

#### 3.1.5. Molecular Diffusion Behavior Analysis

The diffusion coefficient (D) was calculated to evaluate the molecular mobility and rearrangement capability within the HIPEA systems. The calculation methodology for D is outlined as follows:

(2) $$D=\underset{t\to \infty }{\lim}\frac{1}{6Nt}\left({\sum_{i=1}^{N}\left|\mathrm{ri}(t)-r0(t)\right|}^{2}\right)=\frac{k}{6}$$

In the equation, D denotes the diffusion coefficient (Å^2^/ps); N denotes the number of molecules; t denotes the simulated time (ps) and ri(t) denotes the molecule’s position at time t. The parameter k represents the slope of the image with a uniform azimuth shift.

**Figure 12:**
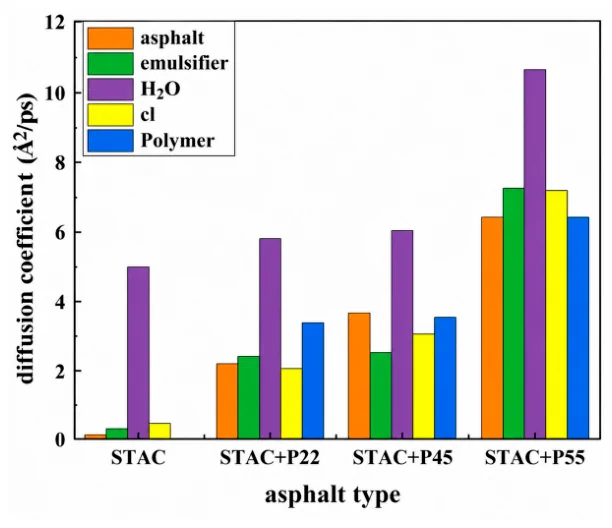
Diffusion coefficients with different EO numbers.

The diffusion coefficients for each constituent of the high-internal-phase emulsified asphalt, as determined by MS analysis, are shown in Figure 12. All systems exhibited a good linear relationship between MSD and simulation time, with R^2^ values exceeding 0.98, confirming the reliability of the fitting results.

Figure 12 indicates that under the studied formulation and preparation conditions, increasing the length of the polyethylene glycol chain significantly enhances the diffusion coefficients of water, emulsifier, and asphalt. The diffusion coefficient of water molecules is the highest, indicating that water molecules have the strongest diffusion ability during the emulsification process. In the pure STAC system, the diffusion coefficients of STAC and asphalt molecules are notably low, indicating limited compatibility and weak interfacial adsorption and interaction. The introduction of polyethylene glycol has substantially altered this scenario. This observation initially appears counterintuitive to the common perception that longer-chain molecules exhibit slower mobility. However, this phenomenon is underpinned by polyethylene glycol chains acting as “molecular bridges,” facilitating robust interactions with water and asphalt (e.g., van der Waals forces, hydrogen bonds). These interactions enhance intercomponent coupling, thereby facilitating the collective movement of the entire molecule. Consequently, the increase in the diffusion coefficient should be interpreted as enhanced interfacial molecular mobility of the composite system rather than the intrinsic self-diffusion of individual PEG chains.

Under the conditions of the formula and preparation method under study, the diffusion coefficient of the polymer increases proportionally with the degree of polymerization. Polymer molecules enhance interfacial interaction by strengthening van der Waals forces between oil and water phases. Simultaneously, facilitated by water and asphalt molecules, polymer chain segments concentrate at the asphalt interface area through molecular diffusion, establishing a stable diffusion layer.

#### 3.1.6. Relative Concentration Distribution Analysis

**Figure 13:**
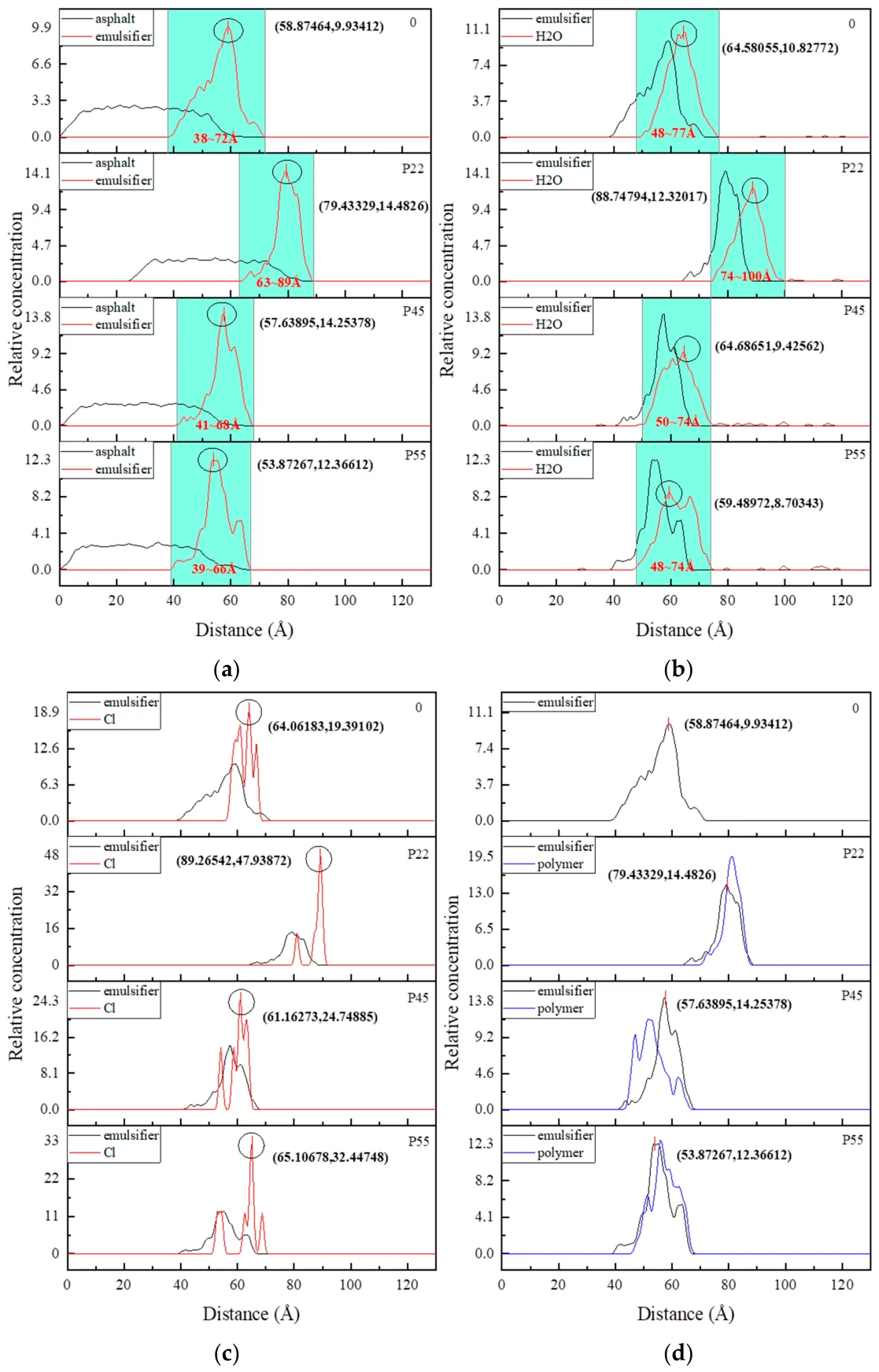
Relative concentration distribution of each component along the Z-axis at 501 ps. (**a**) Asphalt and emulsifier, (**b**) emulsifier and water, (**c**) emulsifier and Cl and (**d**) emulsifier and high polymers.

The spatial distribution of relative concentrations can visually represent the distribution of individual components along the Z-axis within the interface region, thereby indicating the thickness and composition of the interface layer [29,30]. The differences in interface organization may affect the resistance of asphalt droplets to coalescence. However, this study did not directly quantify the interface thickness; therefore, the observed differences in stability are mainly attributed to changes in the interface structure, rather than just the thickness. Consequently, the emulsifier’s interfacial activation intensifies, enhancing interfacial interactions between the emulsifier and the asphalt. As a result, the stability of the emulsified asphalt improves. Figure 13 illustrates the relative concentration distribution of each system at 501 ps.

As depicted in Figure 13a, as the length of the polyethylene glycol chain increases, a higher concentration of STAC molecules is observed between the oil and water phases, driven by van der Waals forces. Some STAC molecules protrude into the spaces among asphalt molecules due to van der Waals interactions between hydrophobic groups and asphalt molecules, thereby promoting their miscibility. Consequently, the adhesive force of STAC on the oil/water molecules at the interface is enhanced, resulting in a more pronounced reduction in interfacial tension between oil and water.

Analysis in Figure 13b reveals that, following the introduction of polyethylene glycol, the distribution of water molecules at the interface becomes more concentrated, indicating that polyethylene glycol more effectively immobilizes water molecules at the interface through interactions between PEG ether oxygen atoms and surrounding water molecules, which may contribute to the interfacial hydration environment., reducing the direct contact between droplets and promoting the stability of droplets.

As depicted in Figure 13c, the Cl ion distribution consistently aligns with the hydrophilic head groups of STAC, validating the presence of electrostatic interactions. The introduction of polyethylene glycol exerts minimal influence on this phenomenon.

Examination of Figure 13d reveals that within the composite system, the concentration distribution profile of polyethylene glycol closely mirrors that of STAC, with nearly identical peak positions. This observation suggests that the polymer, akin to the emulsifier molecules, is situated at the oil–water interface, collaborating with the emulsifiers to enhance their adhesion to oil and water molecules, thereby reducing interfacial tension between the two phases.

### 3.2. Experimental Verification and Analysis

#### 3.2.1. HIPEA Emulsification and Dispersed Phase Characteristics

**Figure 14:**
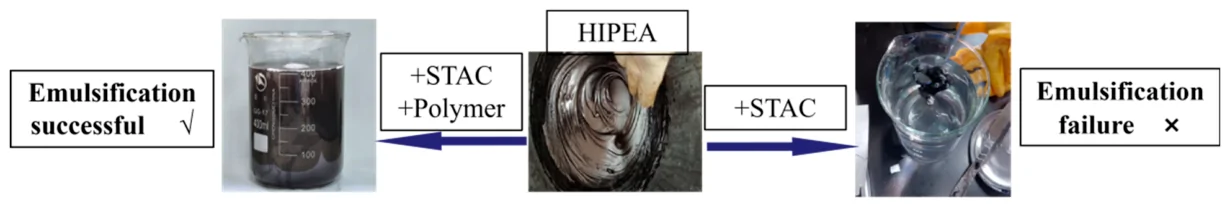
STAC/Polymer emulsification situation.

As shown in Figure 14, the emulsification behavior was assessed by water dispersibility; “x” indicates “Emulsification failure”, while “√” indicates “Emulsification successful”. The STAC-only sample did not form a uniform emulsion under the preparation conditions used [31]. By contrast, the three STAC/PEG samples formed visually homogeneous, paste-like dispersions without obvious macroscopic aggregation. These observations show that PEG addition was associated with successful emulsification in this formulation set; because the simulation compositions were not fully normalized, the result should not be attributed exclusively to EO chain length.

#### 3.2.2. HIPEA Storage Stability Analysis

(1) Analysis of the Evolution of Asphalt Microdroplet Size

**Figure 15:**
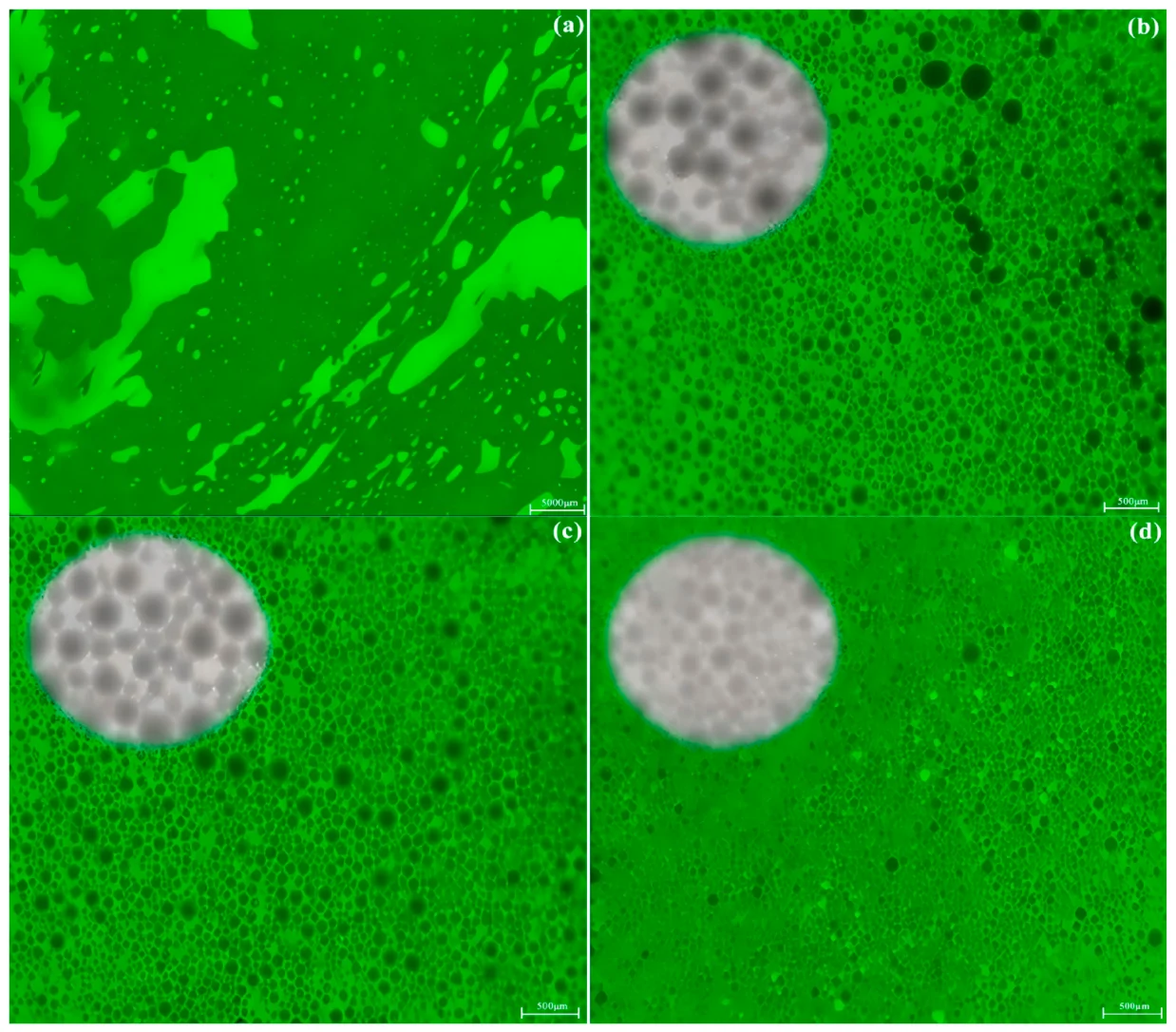
Fluorescence microscope images of fresh emulsions of (**a**) STAC, (**b**) STAC+P22, (**c**) STAC+P45, and (**d**) STAC+P55.

Analysis of droplet size of fresh emulsion. Figure 15 displays fluorescence microscope images revealing that all three HIPEA systems incorporating polyethylene glycol exhibited typical O/W structures, characterized by closely packed asphalt droplets separated solely by a thin water film. In contrast, the STAC system, devoid of polyethylene glycol, underwent oil–water phase separation and failed to achieve emulsification.

**Figure 16:**
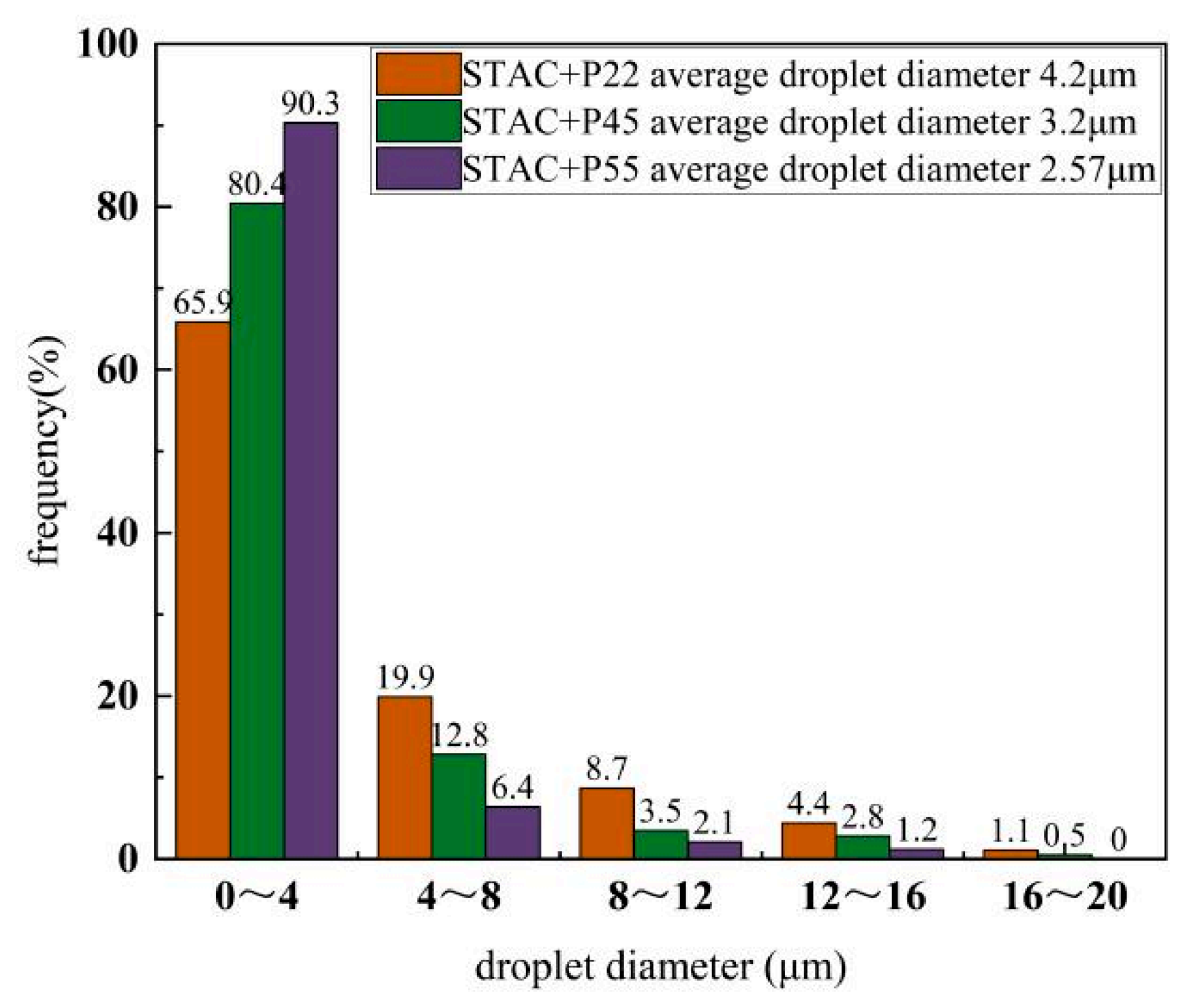
Histogram of particle size distribution of asphalt microdroplets.

The fluorescence microscopic scanning images of three types of successfully emulsified HIPEA were preprocessed using ImageJ 1.54u8. Then, the dispersed phase droplet particles were precisely extracted. Finally, the asphalt droplets were quantitatively analyzed, and the distribution histogram of particle diameters was obtained. The statistical results of the particle diameter distribution are shown in Figure 16. This observation provides direct evidence for the pivotal role of high-molecular-weight polymers in enabling successful HIPEA emulsification. In Figure 16, among the emulsified groups, the STAC+P55 system exhibited the smallest droplet size and the most uniform distribution, with an average particle size of 2.57 μm, surpassing that of STAC+P45 (3.21 μm) and STAC+P22 (4.20 μm), as illustrated. Under the conditions of the studied formula and preparation, as the length of the EO chain in the PEG polymer increases, the droplet distribution within the system becomes more uniform, the particle size of the microdroplets becomes smaller, and the stability of the system improves.

**Figure 17:**
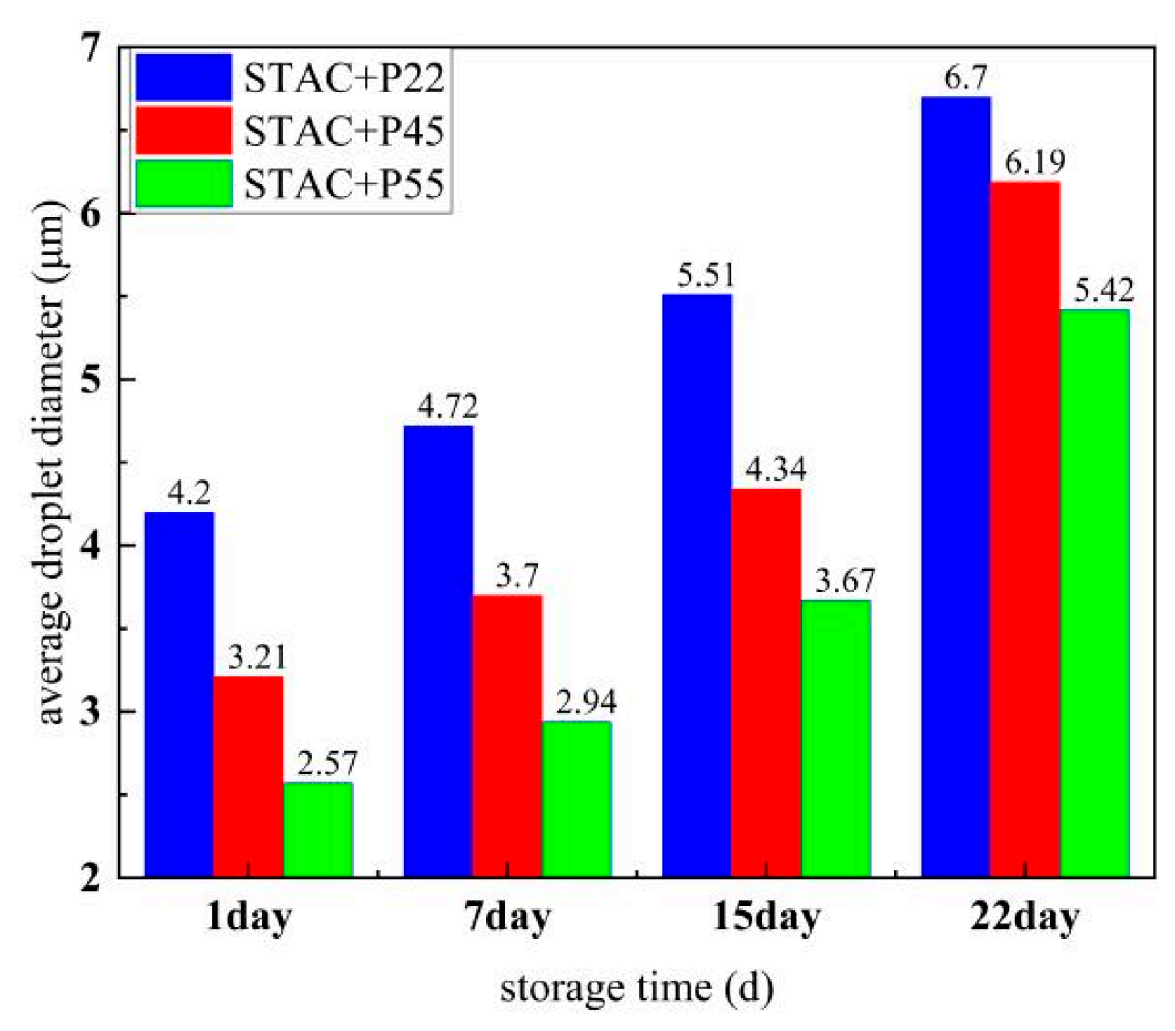
Changes in the average particle size of HIPEA after storage for 1 day, 7 days, 15 days, and 22 days.

Analysis of asphalt droplet evolution during storage. As shown in Figure 17, upon sealing and storing the emulsion for varying durations, it was observed that the droplets in all systems aggregated and coalesced over time, leading to an increase in average particle size, broadened size distribution, and decreased stability. Notably, after 22 days of storage, the average particle size of STAC+P55 (5.42 μm) remained smaller compared to that of STAC+P45 (6.20 μm) and STAC+P22 (6.70 μm). Within the investigated formulation range, increasing the PEG EO number from 22 to 55 effectively improved the resistance of HIPEA to droplet growth during storage.

(2) Dynamic storage stability

**Figure 18:**
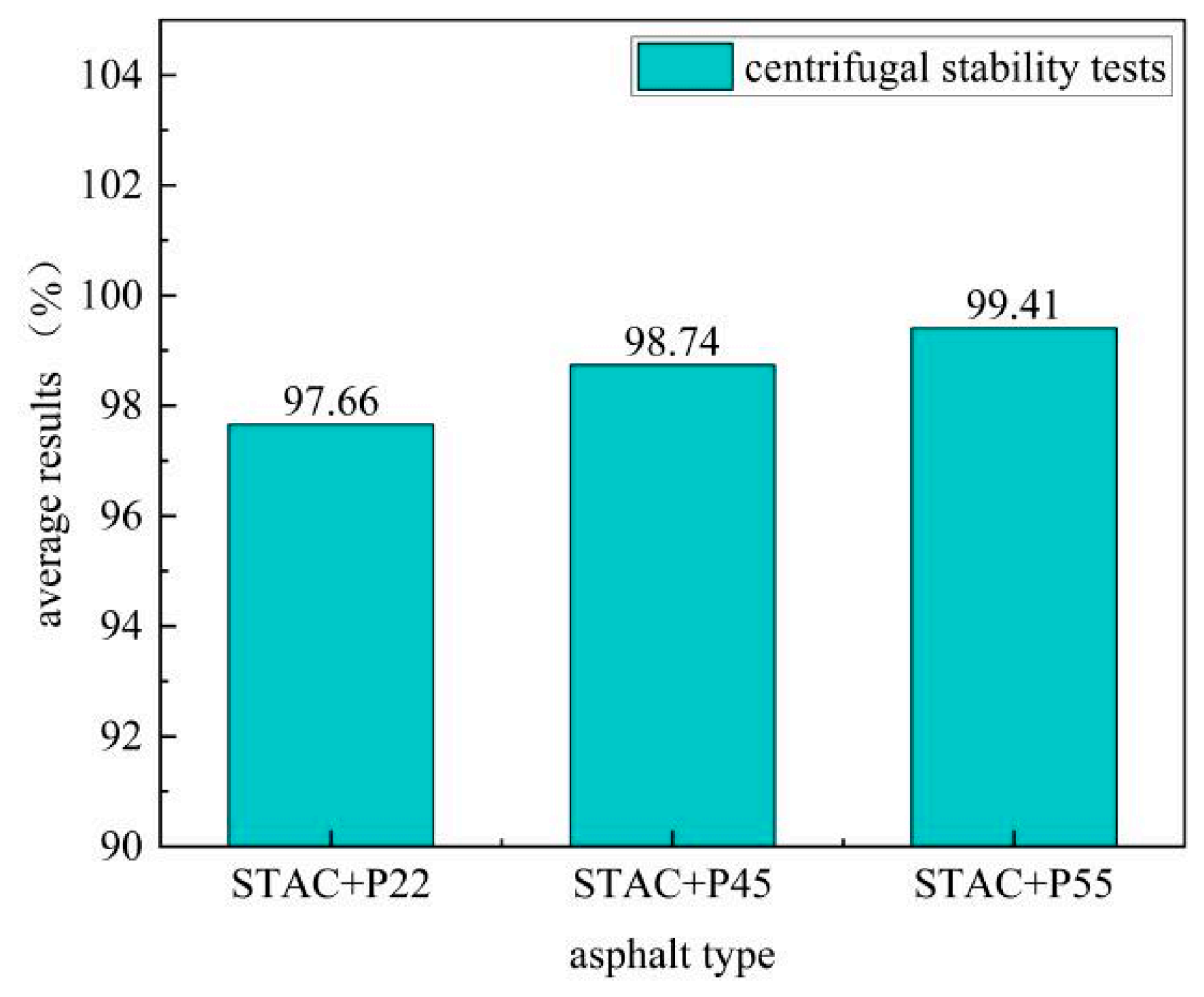
The average data of centrifugal stability of HIPEA.

Centrifugal stability hinges on the interplay between the interfacial energy of emulsions and external forces amid dynamic stress [32]. Higher precipitation of continuous phases under identical centrifugal conditions leads to greater particle-size disparity within the system, diminished interface-mode strength, and heightened system instability [33]. The sequence of mass of the continuous phase in the sedimentation is STAC+P55 (0.059 g) < STAC+P45 (0.126 g) < STAC+P22 (0.233 g), revealing that within the three high-internal-phase emulsifier solution systems, P55 reduced centrifugal phase separation by 74.7% relative to P22. The average centrifugal stability of HIPEA was calculated from the precipitated mass, as shown in Figure 18. These mass values support only a comparison under the reported centrifugation protocol; they do not independently establish the proposed molecular mechanism [34].

#### 3.2.3. HIPEA Thermal Stability Analysis

**Figure 19:**
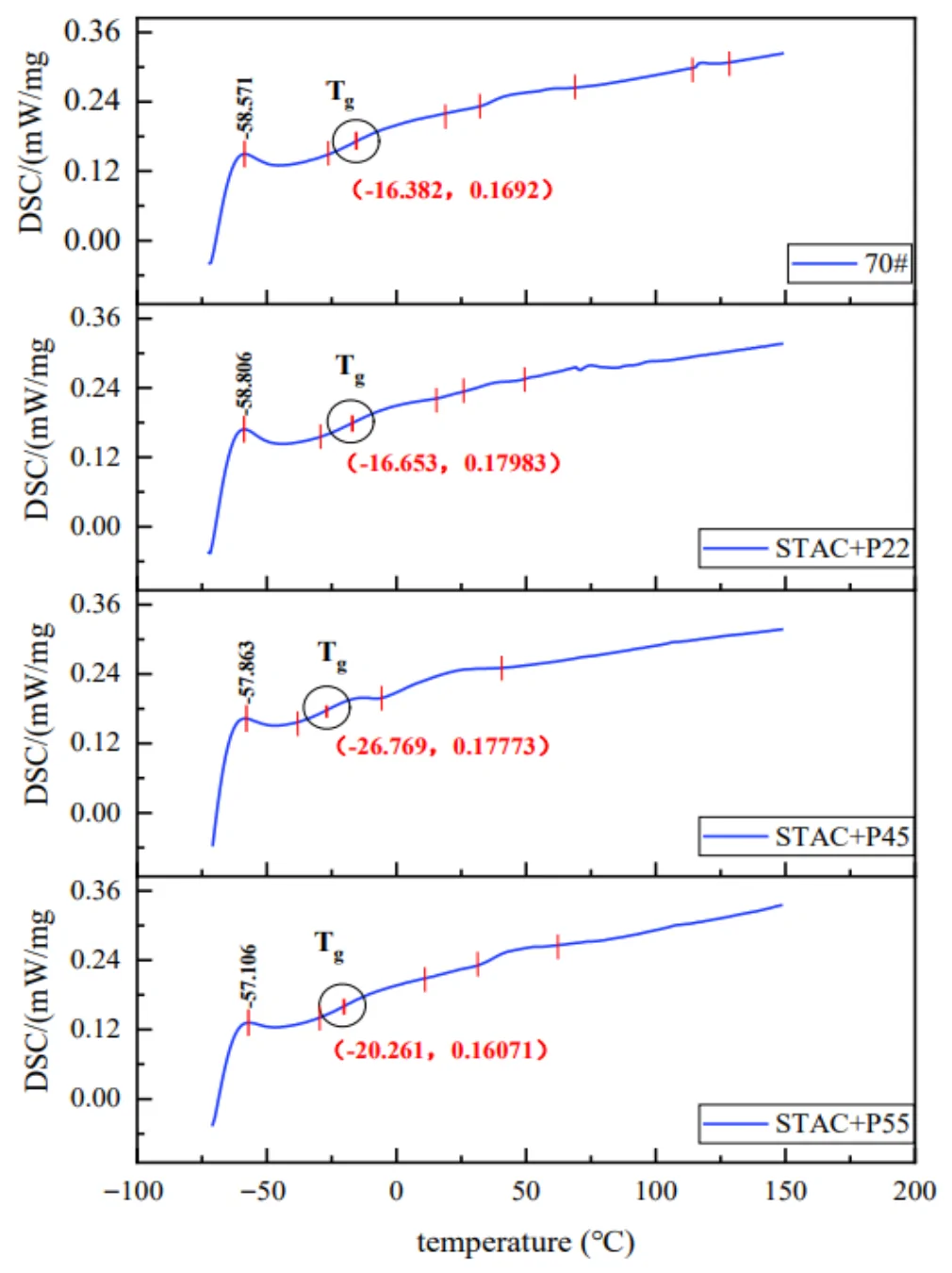
DSC curves of 70# base asphalt and HIPEA.

Differential scanning calorimetry (DSC) was employed to characterize the thermal transition behavior of the base asphalt and HIPEA systems with different PEG chain lengths [35]. The DSC curves and corresponding thermodynamic parameters are presented in Figure 19 and Table 6, respectively. Analysis of the DSC curve and thermodynamic parameters reveals that the 70# matrix asphalt exhibits a distinct endothermic peak around 120 °C. In contrast, the three HIPEA systems show no significant endothermic behavior beyond 70–80 °C. Compared with base asphalt, HIPEA exhibited a lower overall enthalpy response, which can be attributed to its multiphase structure containing water and emulsifying components. Therefore, the DSC enthalpy difference mainly reflects the difference in thermal response between the two systems rather than directly representing thermal stability. A comparative assessment among the three HIPEA formulations reveals the following trend in total ΔH values: STAC+P55 (21.4 J/g) > STAC+P45 (20.6 J/g) > STAC+P22 (11.23 J/g). In DSC analysis of emulsion systems, a higher ΔH indirectly suggests that the internal interfacial film or specific structure requires more energy to be absorbed during transformation [36]. Additionally, the STAC+P55 system exhibits the highest critical temperature (61.105 °C) for the endothermic peak, indicating reduced temperature sensitivity. Among the HIPEA formulations, the increased transition temperature and ΔH values suggest stronger resistance to thermal-induced structural rearrangement, indicating enhanced thermal stability relative to the other formulations.

### 3.3. Cross-Scale Correlation Between Molecular Interactions and Macroscopic Stability

**Figure 20:**
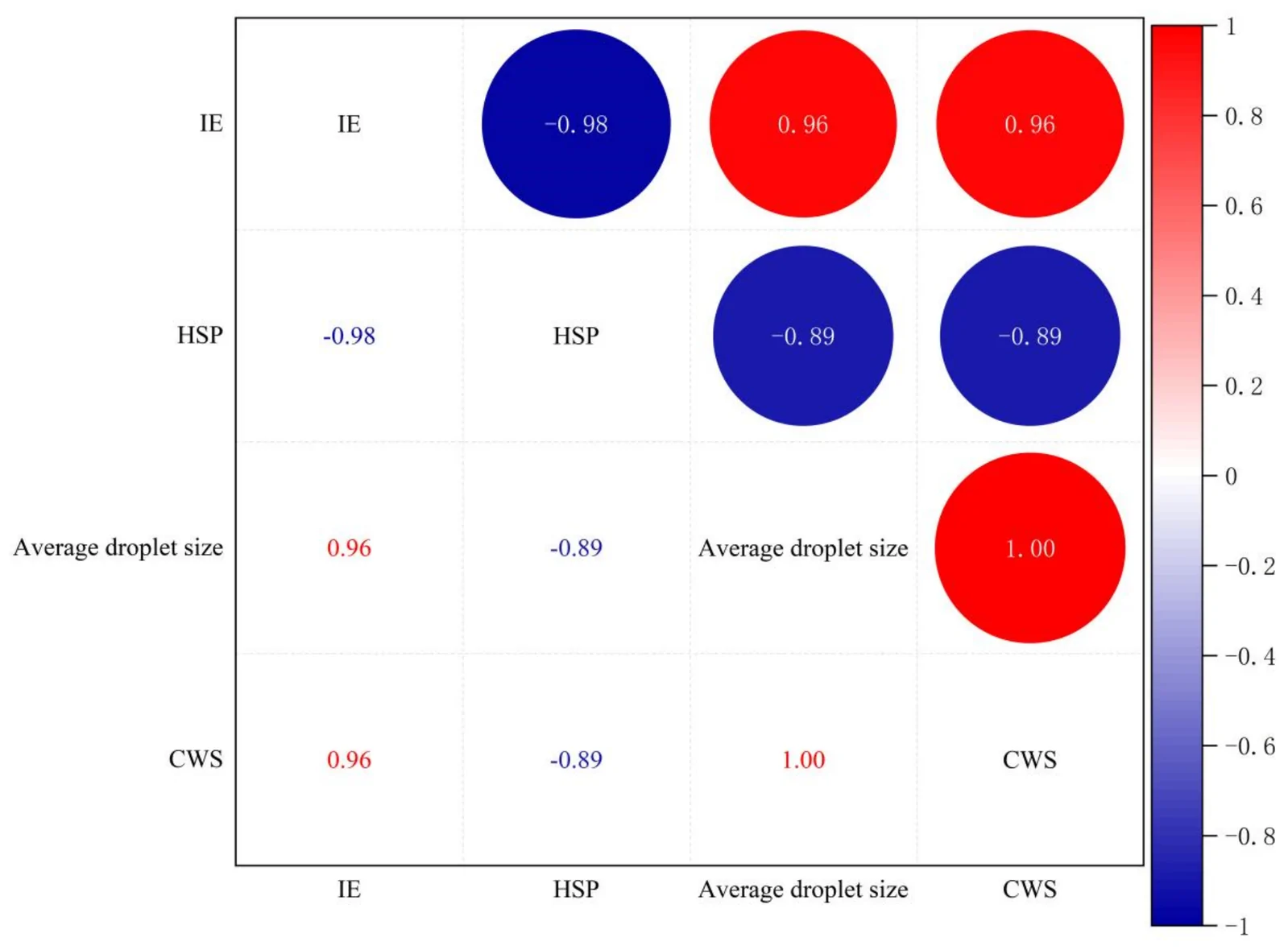
Pearson correlation matrix.

Pearson correlation analysis was performed as an exploratory analysis to evaluate potential relationships between molecular parameters and stability properties among the selected formulations. Due to the limited number of formulations, the obtained correlation coefficients should be regarded as preliminary indicators rather than statistically conclusive relationships. As shown in Figure 20, Pearson correlation analysis was conducted among interfacial formation energy (IE), hydrogen-bond solubility parameter (HSP), average droplet size, and centrifugal water separation (CWS). The calculation method of the Pearson correlation coefficient is shown in Equation (3):

(3) $$r=\frac{\sum_{i=1}^{n}(x_{i}-\bar{x})(y_{i}-\bar{y})}{\sqrt{\sum_{i=1}^{n}(x_{i}-\bar{x}{)}^{2}}\sqrt{\sum_{i=1}^{n}(y_{i}-\bar{y}{)}^{2}}}$$

In the formula, *n* represents the sample size, Xi and yi represent a set of observations for the i-th group, $\bar{x}$ represents the sample mean of x, and $\bar{y}$ represents the sample mean of y. The coefficient ranges from −1 to +1. A positive value indicates a positive linear association, whereas a negative value indicates a negative linear association. A value close to zero indicates a weak linear association, while an absolute value close to one indicates a strong linear association. For descriptive purposes, the absolute magnitude of (r) may be classified approximately as negligible (|r| < 0.20), weak (0.20–0.39), moderate (0.40–0.59), strong (0.60–0.79), or very strong ((|r|\geq0.80)). In this study, the correlation analysis was based on only four formulation systems (*n* = 4). Accordingly, the corresponding degrees of freedom for testing the significance of a Pearson correlation are (*n* − 2 = 2). Given this very limited sample size and the differences in molecular composition among the simulated systems, the calculated coefficients are interpreted as preliminary indicators of linear association rather than statistically conclusive evidence.

As shown in Figure 20, IE exhibits a strong negative correlation with HSP (r = −0.98), demonstrating that interfacial formation energy is closely associated with the molecular interaction characteristics of the system. Meanwhile, HSP shows strong negative correlations with average droplet size and CWS (r = −0.89), indicating that molecular-level interactions are closely related to the evolution of droplet morphology and macroscopic water separation. The strong correlations between IE and both average droplet size and CWS (r = 0.96) further demonstrate that interfacial interaction parameters are closely coupled with macroscopic stability. In the studied formulation, a strong positive correlation was observed between the PEG chain length and the evaluated stability indicators. This result suggests that the PEG chain length may affect the interfacial stability behavior; however, due to the limited number of data points, no clear statistical conclusion can be drawn at present. In future research, I will adopt more independent modeling methods and additional statistical validations to further confirm these correlations.

## 4. Conclusions

This study combined molecular dynamics simulations with laboratory observations to compare STAC-only and STAC/PEG high-internal-phase emulsified asphalt systems. The conclusions are restricted to the formulations and test protocols investigated. First, STAC interacts with PEG at the asphalt–water interface region, and the interaction related to PEG may help to achieve an ordered arrangement of the interface structure and enhance its stability. Molecular dynamics simulations indicate that STAC and PEG interact within the oil–water interfacial region. The PEG chains are distributed in this region and interact with surrounding water molecules, contributing to interfacial hydration and stabilization. Under the tested formulation and preparation conditions, increasing the PEG EO chain length was associated with enhanced interfacial organization and stronger interfacial interactions, with the STAC+P55 system exhibiting the strongest interfacial interaction among the investigated formulations.

Second, in the laboratory dispersion test, the STAC-only formulation did not form a uniform emulsion, whereas STAC+P22, STAC+P45, and STAC+P55 did. For the PEG-containing formulations, the initial mean droplet diameter decreased from 4.20 μm (P22) to 2.57 μm (P55). After 22 days of storage, mean diameters were 6.70, 6.20, and 5.42 μm for P22, P45, and P55, respectively. These results indicate less droplet growth for P55 under the storage conditions used.

Third, after centrifugation at 8000 rpm for 1 h, the separated continuous-phase masses were reported as 0.233, 0.126, and 0.059 g for P22, P45, and P55, respectively; the P22-to-P55 reduction was 74.7%. DSC showed characteristic peak temperatures of 49.853 °C (P22), 51.341 °C (P45), and 61.105 °C (P55). Although the peak shifted to a higher temperature with the tested PEG grade, total enthalpy did not increase monotonically across the formulations, and DSC peak temperature alone cannot establish overall thermal stability.

Finally, Pearson coefficients calculated from only four formulations are exploratory and do not establish statistical significance or causal relationships. The current evidence supports a comparative laboratory assessment of dispersion, storage-related droplet growth, centrifugation-induced separation, and thermal transitions. It does not demonstrate adhesion, rheological performance, curing behavior, residue properties, mixture performance, aging resistance, or field pavement performance. Future work should include additional independent MD simulations, composition-normalized models, and further statistical validation to examine the robustness of the observed relationships.

## Figures and Tables

**Table 1:** Technical indicators of Kunlun 70# base asphalt.

Item	Penetration (0.1 mm/25 °C)	Softening Point (℃)	15 °C Ductility (cm)
Kunlun 70# base asphalt	72	46	>150

**Table 2:** Proportions of the four SARA fractions in Kunlun 70# base asphalt.

Bitumen Components	Asphaltenes	Resins	Aromatics	Saturates
Proportion (%)	12.44	26.62	44.62	16.32

**Table 3:** The number of molecules added in each of the four asphalt systems.

System	STAC	STAC+P22	STAC+P45	STAC+P55
N_asphalt molecules_	3	3	3	3
N_STAC_	12	6	6	7
N_Polymer_	0	2	1	1
N_H2O_	500	500	500	450

**Table 4:** Composition and mass ratio of test materials.

System	Kunlun 70# Base Asphalt	WATER	STAC	PEG
STAC	80	16	4	0
STAC+P22/P45/P55	80	16	2	2

**Table 5:** Interfacial formation energy of four high-internal-phase emulsified asphalts.

Systems	*E*_Total_	*E*_Asp_	*E*_Emul_	Δ*E*
STAC	−268.956	3234.359	−3260.450	−242.870
STAC+P22	477.887	3525.281	−2791.130	−256.262
STAC+P45	394.639	3393.565	−2715.890	−283.034
STAC+P55	784.794	3402.403	−2291.250	−326.359

**Table 6:** Thermodynamic parameter data of 70# base asphalt and HIPEA.

Asphalt Type	T_g_ (°C)	Peak Temperature (°C)	Total ΔH (J/g)
70#asphalt	−16.382	18.936/68.936/128.936	23.650
STAC+P22	−16.653	15.853/49.853/	11.230
STAC+P45	−26.769	−6.863/51.341	20.600
STAC+P55	−20.261	10.105/61.105	21.400

## Data Availability

The original contributions presented in the study are included in the article; further inquiries can be directed to the corresponding author.
